# The Prospective Advantages of Baltimore as a Medical Centre

**Published:** 1880-06

**Authors:** 


The Prospective Advantages of Baltimore as a Medical Centre are quite clearly pre-figured in a re-print by Dr. John Van Bibber, of this city, now before us. It is our purpose to direct the attention of our readers to the subjects he discusses at some length, as they approach more nearly their consummation. The *Johns Hopkins Hospital* is rapidly advancing toward completion, and when that is attained and it is fully equipped for its work, it will really be without a peer in the world. The advantages which it will be capable of offering *all* who might wish to avail themselves of its vast resources must draw large numbers of students from every quarter, should it be conducted in the spirit of the testator.

*The Wilson Sanitarium, for Children of Baltimore City*, will be another noble charity. It is abundantly endowed and will be altogether unique in aim and operation. Probably no other institution in existence will, or ought to compare with it, in the field it is to occupy, when completed in all its appointments

*The Sheppard Asylum*, for the treatment of the insane, will be a model worthy of imitation when it is fully into its work. It will soon cause the name of its munificent founder to take rank, not only with the illustrious and great philanthropists, who have done so much for Baltimore, but amongst those of the world as well.

